# From Observation to Action: Improving Staff Confidence in Identifying and Escalating Physical Health Deterioration in an Older Adult Mental Health service

**DOI:** 10.1192/bjo.2026.11312

**Published:** 2026-06-30

**Authors:** Scout Irving, Lian David, Kunle Ashaye

**Affiliations:** Hertfordshire Partnership NHS Trust, Hertfordshire, United Kingdom

## Abstract

**Aims:**

People with severe mental health problems are affected by disparities in physical health outcomes compared to the general population. The risk of physical deterioration increases as patients grow older and comorbidities accumulate. This risk is particularly relevant in the population affected by functional and cognitive psychiatric disorders, where patients’ ability to detect and report symptoms can be impaired. This places a key responsibility on staff in older adult mental health services, who are well positioned to identify deterioration through routine physical health monitoring. However, confidence gaps exist amongst multidisciplinary team members in the recognition and escalation of physical health deterioration. To address this, the physical health teaching programme was implemented.

Hypothesis: The teaching programme will result in an improvement in the perceived confidence of staff in identifying physical health deterioration and escalating concerns.

**Methods:**

Weekly teaching sessions were conducted in an older adult mental health service over a period of three months. All members of the multidisciplinary team across both inpatient and community settings were invited to participate. Teaching was delivered using posters and interactive worksheets, covering topics such as vital signs, fluid intake, bowel output monitoring and delirium. Pre- and post-teaching questionnaires were designed to evaluate confidence levels using a Likert scale and knowledge was assessed via multiple-choice questions.

**Results:**

Results were analysed from 17 participants who completed both pre- and post-teaching questionnaires. The first two teaching sessions focused on vital signs and fluid intake. Preliminary findings demonstrated an overall improvement in staff confidence. However, pre-teaching knowledge scores were high for these topics and minimal improvement was seen on repeating the multiple-choice questions. These findings demonstrate the feasibility of a targeted physical health teaching programme.

**Conclusion:**

A physical health teaching programme can improve staff confidence in identifying and escalating physical health deterioration in an older adult mental health service.

